# Artificial Intelligence in Telemedicine: philosophical, ethical, and epistemological dimensions of a transforming practice

**DOI:** 10.31744/einstein_journal/2026AE2371

**Published:** 2026-09-15

**Authors:** Agnaldo Rodrigues da Costa, Carlos Henrique Sartorato Pedrotti, Tarso Augusto Duenhas Accorsi

**Affiliations:** 1 Hospital Israelita Albert Einstein São Paulo SP Brazil Hospital Israelita Albert Einstein, São Paulo, SP, Brazil.

**Keywords:** Artificial Intelligence, Telemedicine, Medical ethics, Knowledge, Philosophy, Attitude of health personnel, Healthcare delivery

## Abstract

In the aftermath of the COVID-19 pandemic, telemedicine has evolved from a contingency-based alternative into a structural pillar of healthcare delivery, increasingly being integrated with artificial intelligence. Algorithms for triage, clinical decision support, remote monitoring, and generative language models now actively participate in medical care. This integration is more than just functional; it reorganizes the foundational aspects of medicine—ontological, ethical, and epistemological—by redefining what qualifies as valid knowledge, who has legitimate authority, and how care is organized when an algorithmic agent facilitates the clinical interaction. This article critically examines the emergence of the patient-clinician-algorithm triad in telemedicine, and analyzes its implications for autonomy, informed consent, epistemic trust, privacy, justice, responsibility, and the phenomenology of care. It argues that the future of medicine will not be human or algorithmic but human-algorithmic, provided clinical judgment, explainability, and moral responsibility remain central.

## Highlights

■ Artificial Intelligence transforms telemedicine from a doctor-patient dyad to a complex three-way triad.

■ Clinical judgment must remain the final authority despite algorithmic power.

■ Shared responsibility is split between clinicians, institutions, and developers.

■ Explainability is a fundamental requirement for ethical medical actions.

## In Brief

This paper analyzes the role of a patient-clinician-algorithm triad in Telemedicine. It examines how Artificial Intelligence reconfigures medical authority and responsibility, arguing that while Artificial Intelligence offers diagnostic power, medical practice must remain human-centered. It proposes frameworks for shared responsibility, informed consent, and fairness.

## Graphical abstract

**Figure:**
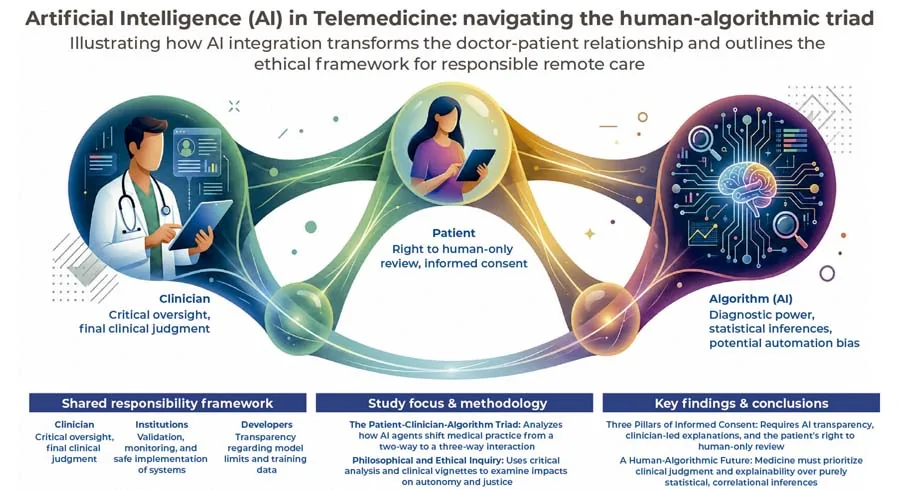

## INTRODUCTION

The integration of Artificial Intelligence (AI) into Telemedicine (TM) decisively accelerated after the COVID-19 pandemic, representing not merely an incremental technological innovation but a structural transformation of medical practice itself. Remote care has ceased to function as a simple alternative access channel and has become the primary site of convergence between medicine and algorithmic systems capable of triaging symptoms, suggesting diagnoses, monitoring patients at a distance, and producing clinical narratives that simulate medical reasoning. In this evolving landscape, the traditional physician-patient relationship has shifted from a dyadic to a more stable triad, where the algorithm—lacking embodiment and moral intent—holds significant inferential power and impacts decisions that directly affect human lives.^(1)^

This algorithmic presence is not neutral. It reconfigures the ontology of medical practice by shifting the loci of knowledge, authority, and responsibility. Clinical knowledge, traditionally grounded in causal narratives, physical examination, and contextual judgment, now coexists with statistical rationality based on high-dimensional correlations. Deep learning models perform comparably to or better than human experts in specific domains such as dermatology, ophthalmology, and radiology, challenging established epistemic hierarchies.^(2)^ However, these improvements in accuracy come with a lack of transparency. The so-called algorithmic “black box” often makes it difficult for clinicians to understand the reasons behind a recommendation in clear terms. Consequently, epistemic authority becomes uncertain, as trusting the system increasingly relies on statistical predictions derived from abstract populations rather than on the unique details of the patient visible on the TM screen.^(3)^

This opacity has direct implications for autonomy and informed consent. When clinicians cannot explain why a system has recommended a particular course of action, consent risks becoming performative-formally obtained but substantively hollow. The problem intensifies in contexts of vulnerability, such as pediatric care, older adults with cognitive impairment, or severely ill patients, where continuous-learning systems may alter clinical decisions over time without renewed consent. Documented cases of automated prescribing or unsupervised recommendations resulting in serious adverse outcomes illustrate the risk of eroding human agency in the name of efficiency. Preserving autonomy in this context requires not only transparency about AI use but also mandatory human oversight, the right to refuse, and the retention of clinical judgment as the final decision-making authority.^(4)^

### A clinical vignette: algorithmic triage and moral residue in remote care

To illustrate the ethical and epistemological tensions described above, consider the following hypothetical scenario.

A 62-year-old woman with a history of diabetes and hypertension initiates a teleconsultation, reporting chest discomfort and fatigue. Before interacting with the physician, she is first assessed by an AI-driven triage system embedded in the TM platform. Based on probabilistic modeling trained largely on datasets dominated by younger populations, the system classifies her symptoms as “low cardiovascular risk” and recommends outpatient monitoring rather than urgent referral. The physician, reassured by the algorithmic output and operating under time pressure within a high-volume TM service, initially follows the recommendation. Several hours later, however, the patient presents to a local emergency department with an evolving myocardial infarction.

This vignette illustrates how epistemic authority may subtly shift from clinician to algorithm in AI-mediated care.^(5)^ The ethical tension arises not from malicious intent but from the structural features of algorithmic systems: opacity, statistical generalization, and automation bias. In this context, the patient’s consent rarely encompasses probabilistic stratification performed by opaque models, and clinicians themselves may lack the epistemic tools needed to critically interrogate algorithmic outputs.^(6)^

Responsibility becomes distributed among multiple actors: in this case, the clinician who relied on the recommendation, the institution that implemented the system, and the developers who trained it on potentially non-representative datasets. The example demonstrates that the ethical challenge of AI in TM is not merely the possibility of error, but the way in which such errors are structured, legitimized, and socially diffused.^(7)^

Algorithmic mediation also reshapes the ethics of care by altering the lived experience of the physician-patient relationship. Telemedicine had already diminished the corporeality of the clinical encounter; the introduction of AI deepens this distance by replacing elements of listening, interpretation, and shared silence with automated recommendations. There is a risk of commodifying interaction, in which care becomes a data stream and human suffering is translated into monitorable variables. At the same time, proponents of reflective integration argue that automating routine tasks can free time and cognitive resources for more empathic, patient-centered care. This potential, however, can only be realized if algorithms are integrated as explicable support tools for clinical reasoning rather than as substitutes for human deliberation.

Ethical concerns further expand when considering the centrality of data. AI-enabled TM depends on continuous flows of sensitive information collected through digital platforms and wearable devices. Consent, often buried within lengthy and opaque terms of service, rarely reflects a genuinely informed choice. Data breaches, security failures, and unauthorized uses erode public trust and cause irreversible harms, as health information touches the core of personal intimacy. Technical strategies such as federated learning, decentralized architectures, and audit logs are frequently proposed as mitigations, but they are effective only when embedded within robust ethical governance and institutional accountability.^(8)^

### Regulatory landscapes: comparative perspectives

Regulatory approaches to AI in healthcare are rapidly evolving across different regions.^(9)^ In the European Union, governance is guided by the General Data Protection Regulation (GDPR) and the upcoming Artificial Intelligence Act, which focus on transparency, risk classification, and the right to explanation in automated decision-making.

In the United States, the Food and Drug Administration (FDA) oversees AI-enabled medical devices through adaptable frameworks designed to handle continuously learning algorithms, with particular attention to safety, effectiveness, and post-market surveillance.

In Brazil, the General Data Protection Law (LGPD *- Lei Geral de Proteção de Dados*) establishes principles of transparency, necessity, and accountability in processing health data. Meanwhile, ANVISA (*Agência Nacional de Vigilância Sanitária*, the Brazilian Health Regulatory Agency) is increasingly assessing AI-based clinical software within current regulatory pathways.^(10)^

Although there are differences in institutional design, these regulatory frameworks share core principles: transparency, human oversight, and institutional accountability.

### Normative proposals for ethical integration

#### Informed consent in AI-mediated Telemedicine

Ensuring meaningful patient autonomy in AI-enabled TM requires an informed consent model that reflects how technology mediates clinical decisions. At a minimum, three elements should be included.^(11)^

First, patients must be clearly informed when algorithmic systems are involved in triage, diagnostic support, clinical documentation, or risk stratification. Transparency about AI system involvement is essential for meaningful consent. Second, clinicians should be able to provide clear explanations of how algorithmic recommendations influence clinical reasoning, even when full technical transparency isn’t possible. Without such explanations, consent risks becoming merely procedural instead of truly informed. Third, patients should have the right to request that clinical decisions be reviewed solely by humans, without it affecting the quality or availability of care. Collectively, these measures help ensure that informed consent remains ethically significant in technologically mediated healthcare settings.

## Mitigating algorithmic bias and digital discrimination

Algorithmic fairness cannot be assumed; it must be demonstrated through evidence. Artificial Intelligence systems trained on datasets that lack sufficient representation of demographic diversity may replicate or even increase existing health disparities.^(12)^Therefore, reducing digital discrimination requires multiple complementary strategies. Training datasets should include demographic, geographic, and socioeconomic diversity. Ongoing post-deployment audits are essential to detect systematic disparities in algorithmic performance across different populations. Additionally, TM initiatives must be supported by investments in digital infrastructure and technological literacy, especially in underserved regions. Without these structural measures, innovations meant to improve healthcare access may inadvertently reinforce existing inequalities.

## Responsibility and accountability

Responsibility in AI-assisted TM must stay rooted in human moral agency. Although algorithms can influence clinical reasoning, they lack intentionality, accountability, and ethical judgment.^(13)^

A shared responsibility framework is therefore essential. Clinicians retain the duty of critical oversight and remain responsible for the final clinical decisions. Healthcare institutions are responsible for validating, monitoring, and safely implementing AI systems in clinical settings. Developers and technology providers must be transparent about model limitations, training datasets, and performance details. Maintaining this human-centered accountability framework is vital for maintaining trust in AI-supported healthcare.

The promise of expanded access also warrants scrutiny. In rural or low-income settings, algorithmic systems can bring patients closer to previously inaccessible clinical experts. Yet these benefits depend on digital infrastructure, connectivity, technological literacy, and cultural adaptation. Without these conditions, innovation risks deepening preexisting inequalities. Algorithmic biases stemming from homogeneous training datasets reproduce structural injustices and lead to systematic diagnostic errors in underrepresented populations. Responsibility for AI-mediated errors becomes diffused among clinicians, institutions, and developers, underscoring the need for regulatory frameworks that ensure accountability.

To prevent digital discrimination, it is recommended to implement complementary strategies.^(14)^ First, training datasets must reflect demographic diversity, incorporating variation in age, ethnicity, socioeconomic status, and geographic context. Second, continuous post-deployment auditing should be implemented to identify systematic disparities in algorithmic performance across different populations. Third, TM programs should be accompanied by investments in digital infrastructure and technological literacy, particularly in underserved regions, to prevent AI-enabled care from inadvertently reinforcing existing structural inequalities. These measures aim to ensure that algorithmic innovation contributes to expanding, rather than restricting, equitable access to healthcare.

We propose a shared responsibility framework involving three actors toward the responsibility for incorrect diagnoses:^(15)^

- Clinicians, who retain the obligation of critical oversight and remain responsible for final clinical judgment;
- Healthcare institutions, which are responsible for the validation, safe implementation, and monitoring of AI systems used in clinical practice;
- Developers and technology providers, who must ensure transparency regarding model limitations, training data characteristics, and system performance.

Although AI systems may influence medical decisions, they do not possess moral agency. Ethical and legal responsibility must therefore remain anchored in human actors and institutional governance structures.

From an epistemological perspective, AI-mediated medicine increasingly shifts from causal explanation to correlational inference, requiring continuous pragmatic validation in real-world settings. This transition risks epistemic injustice by reducing singular narratives of suffering to statistical outliers or noise. At the same time, cognitive offloading threatens clinical critical thinking by fostering excessive reliance on automated recommendations. Addressing these risks requires epistemic humility, algorithmic literacy, and ethical verification mechanisms that preserve the capacity to question the system.^(16)^

In this context, the demand for explainability shifts from a technical detail to a fundamental requirement for the legitimacy of medical actions. Without explanations that can be shared and understood, true ethical reflection and informed decisions become unfeasible. Although generative language models are fluent and coherent, they exhibit critical flaws, including a lack of embodied understanding of suffering, a moral imagination contextualized in real situations, and a sense of responsibility. Medicine is therefore at a pivotal point in history. The future will not be solely human or purely algorithmic but a blend of both—so long as clinical practice maintains the ability to ask why a recommendation was made, who truly makes the decisions, and whom the system ultimately benefits.
